# Enhanced IL-34 expression in Nivolumab-resistant metastatic melanoma

**DOI:** 10.1186/s41232-018-0060-2

**Published:** 2018-03-05

**Authors:** Nanumi Han, Muhammad Baghdadi, Kozo Ishikawa, Hiraku Endo, Takuto Kobayashi, Haruka Wada, Keisuke Imafuku, Hiroo Hata, Ken-ichiro Seino

**Affiliations:** 1 Division of Immunobiology, Institute for Genetic Medicine, Hokkaido University, Kita-15 Nishi-7, Sapporo, 060-0815 Japan; 2 Department of Dermatology, Hokkaido University Graduate School of Medicine, Kita-15 Nishi-7, Sapporo, 060-8638 Japan

**Keywords:** Interleukin-34, Immunotherapy, Melanoma, Tumor-associated macrophage

## Abstract

**Background:**

Immunotherapies that target immune-checkpoint molecules such PD-1 have helped to achieve durable responses in melanoma treatment. However, 25% of melanoma patients who showed objective responses to PD-1 blockade develop resistance and suffer from disease progression and ultimately death, which necessitates the identification of related resistance mechanisms.

IL-34 is a cytokine that controls the biology of myeloid cell lineage through binding to CSF-1R. IL-34 is importantly involved in the pathogenesis of various diseases. In cancer, the expression of IL-34 has been suggested to associate with tumor growth, metastasis, angiogenesis, and therapeutic resistance such as in lung cancers and malignant pleural mesotheliomas. In this study, we evaluate the possible involvement of IL-34 in immunotherapeutic resistance.

**Case presentation:**

Melanoma resection species were obtained from a patient who developed a refractory melanoma against immunotherapy with Nivolumab, and stained with anti-IL-34, anti-melanoma antigens and anti-CD163 antibody. Staining of these markers was compared between primary or metastatic refractory melanoma tissues. Immunohistochemistry staining of melanoma tissues showed an enhanced expression of IL-34 in metastatic refractory melanoma compared to primary melanoma tissues, which correlates with increased frequencies of CD163^+^ macrophages.

**Conclusion:**

We introduce for the first time a clinical case of a patient with metastatic refractory melanoma that acquired resistance to anti-PD-1 immunotherapy, showing an enhanced expression of IL-34 in refractory melanoma tissues.

## Background

Durable responses in melanoma treatment have been achieved with immunotherapies that target immune-checkpoint molecules such as CTLA4 [1–3] and PD-1 [4, 5]. However, 25% of melanoma patients who showed objective responses to PD-1 blockade develop resistance and suffer from disease progression at a median follow-up of 21 months [6]. The mechanisms of immune-resistance in melanoma remain largely unknown. Previous studies suggested a correlation between genetic mutations and acquired resistance to immunotherapy [7–10], such as the loss of beta-2-microglobulin [11] or defects in the interferon signaling pathways [12]. Additionally, tumor cells-derived factors were shown to play critical roles in modifying the complex network between tumor and non-tumor cells at the tumor microenvironment which importantly contributes to therapeutic resistance [13]. For example, gliomas sensitivity to CSF-1R inhibition were found to be importantly impacted by the expression of IGF-1 and IL-4 at the tumor microenvironment in a model of GBM [14]. Obviously, determining the characteristics of therapeutic-resistant tumors is the key to overcome resistance problem in cancer therapy.

IL-34 is a unique cytokine that controls the biology of myeloid cell lineage such as monocytes, macrophages and osteoclasts through binding to CSF-1R [15]. IL-34 expression is restricted under physiological conditions to skin and brain, where it controls the development, biology and function of Langerhans cells and microglia, respectively [16, 17]. In addition to its physiological functions, IL-34 is importantly involved in the pathogenesis of various diseases including autoimmune diseases, inflammation, infections, metabolic disorders, and cancer [17]. In cancer, the expression of IL-34 has been suggested to associate with tumor growth, metastasis, angiogenesis and importantly to therapeutic resistance such as in lung cancers and malignant pleural mesotheliomas [18–24]. Consist with these pro-tumorigenic functions, IL-34 expression was found to correlate with disease progression and poor prognosis in several cancers [25, 26]. In this study, we evaluate the possible involvement of IL-34 in immunotherapy resistance of melanomas. We introduce for the first time a case of patient with refractory malignant melanoma that acquired therapeutic resistance after several rounds of chemotherapy and Nivolumab-based immunotherapy, and compare the expression levels of IL-34 between primary or refractory Nivolumab-resistant metastatic melanoma.

## Case presentation

The patient in this study was diagnosed on February 2008 with melanoma by the Department of Dermatology (Hokkaido University Hospital, Sapporo) based on clinical presentation and histologic examination. This patient is a 74-year-old Japanese woman presented with a history of a 5-mm thickness pigmented lesion on the sole of the left foot and underwent operative surgery. A diagnosis of Stage III B melanoma was confirmed postoperatively by pathological examination, and classified as pT4bN2aM0 according to Melanoma TNM classification. The patient was then treated with interferon β (DAV-Feron), which was stopped after the first dose at the patient’s request. In 2014, the patient was presented again with in-transit metastases, and was treated with Dacarbazine (8 doses) between 2014 and 2015. After the 8th dose of Dacarbazine, metastatic melanoma was identified at distant lymph nodes. The patient was then treated with Nivolumab (2 mg/kg/3 weeks) between 2015 and 2016. Response evaluation criteria showed a progressive disease (PD) on the 4th dose, and changed into a partial response (PR) on the 15th dose. However, the patient showed enhanced melanoma metastasis to another distant lymph nodes on the same thigh, and sampling was performed on December 2016 on the new metastatic sites. The patients’ survival was confirmed on August 2017.

Melanoma resection species were obtained from the patient, and utilized to compare IL-34 expression between primary and metastatic refractory melanoma. Multiplex immunofluorescent staining was performed using Opal 4-color fluorescent IHC kit (Perkin-Elmer NEL810001KT). In details, 4-μm thin sections of paraffin-embedded clinical specimens derived from primary or metastatic melanoma tissues were de-paraffinized in xylene and rehydrated in ethanol. Antigen retrieval was carried out using Immunosavers® (Nissin, Tokyo, Japan) at 98 °C for 45 min. To reduce endogenous peroxidase activities, sections were treated with 0.3% H_2_O_2_ at room temperature for 10 min. After blocking by 4% Block Ace (DS Pharma Biomedical, Tokyo, Japan), sections were incubated with primary antibodies as follows: anti-human IL-34 antibody (1:200 dilution, clone 1D12, Millipore, MABT493), anti-human melanoma antibody (1:50 dilution, Abcam ab732), anti-human CD163 antibody (1:200 dilution, BIO-RAD MCA1853T) at 4 °C overnight. Then, sections were washed by PBS-T (0.1 M PBS with 0.3% Triton) for three times. Moreover, after blocking with 10% goat normal serum, sections were incubated with HRP-conjugated goat anti-mouse IgG antibody (1:100 dilution, BioLegend 405,306) at room temperature for 30 min. After washing by PBS-T (0.1 M PBS with 0.3% Triton) for three times, sections were immersed by fluorophore amplification reagent for 10 min. Finally, sections were counterstained with DAPI for 5 min and mounted in VECTASHIELD mounting medium. Tumor areas were objectively judged by two independent researchers at 600× magnification for each section, and quantification of IL-34, melanoma antigens or CD163 immunoreactivity on the randomly-selected more than 20 tumor areas in each section was measured using FV1000 OLYMPUS software.

**Fig. 1:**
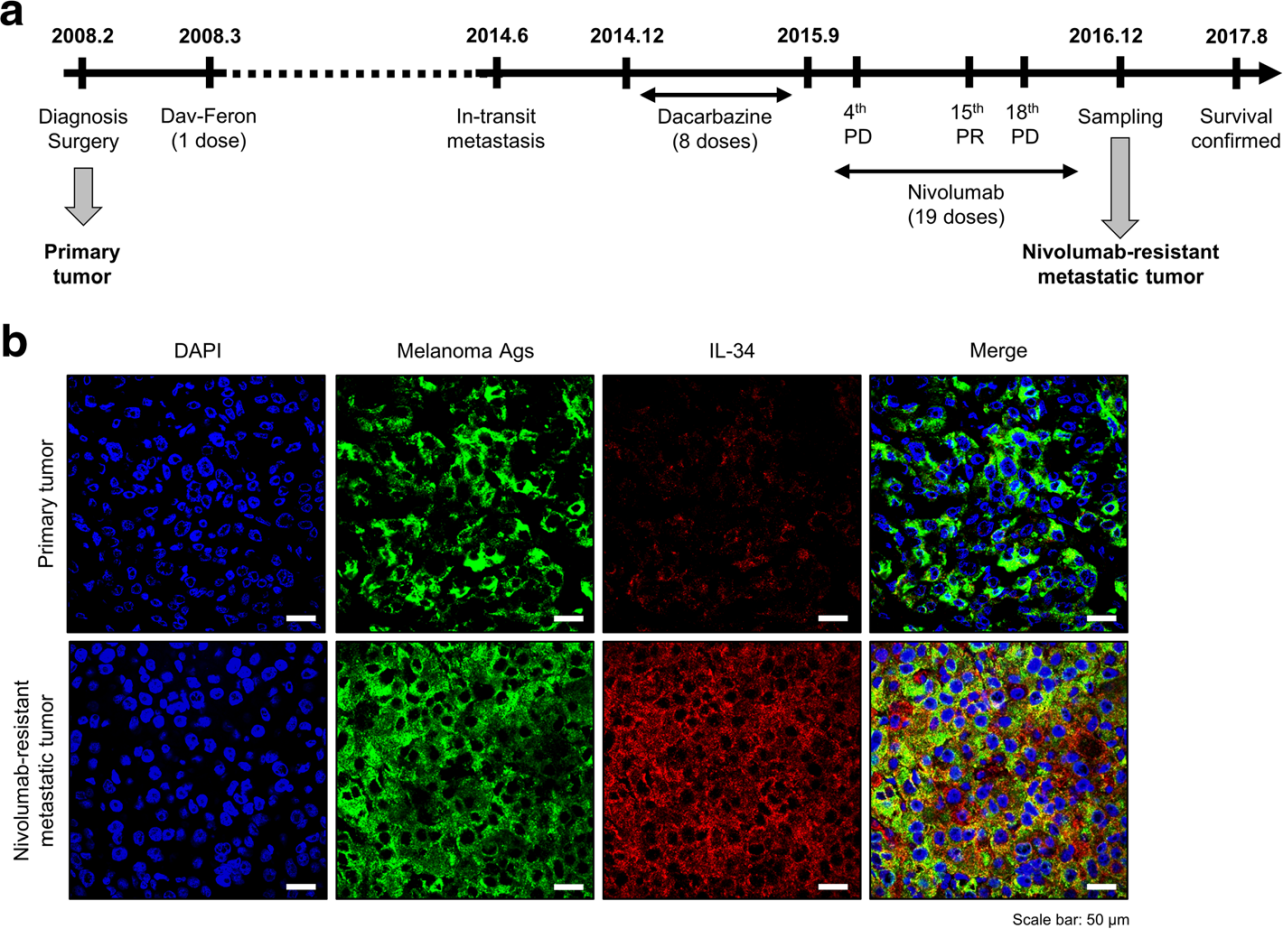
IL-34 expression in Nivolumab-resistant metastatic melanoma. (**a**) Scheme showing the chronologic progression of disease and treatment procedures. (**b**) Representative data of immunohistochemistry staining of IL-34 or melanoma antigens in primary or Nivolumab-resistant metastatic melanoma

**Fig. 2:**
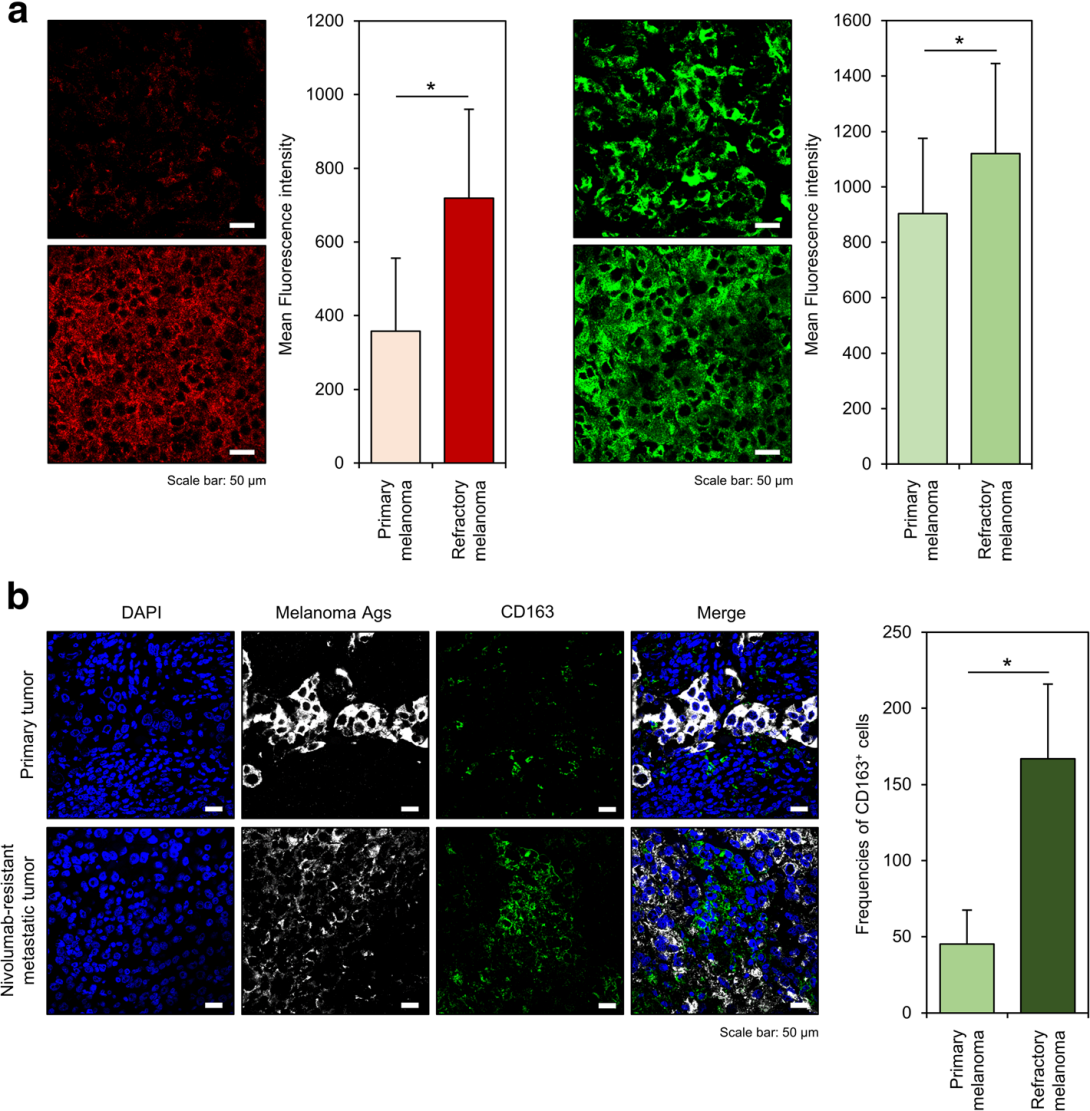
Enhanced IL-34 expression in Nivolumab-resistant metastatic melanoma correlates with increased numbers of infiltrating CD163^+^ cells. (**a**) Representative data of immunohistochemistry staining of IL-34 (left) or melanoma antigens (right) was compared between primary or Nivolumab-resistant metastatic melanoma tissues. Bar graph on the right shows mean fluorescence intensity of IL-34 or melanoma antigens staining in tumor areas. (**b**) Immunohistochemistry staining of CD163 was compared between primary or Nivolumab-resistant metastatic melanoma tissues. Bar graph on the right shows frequencies of CD163^+^ cells in the indicated samples. Data is shown as mean ± SEM. *P < 0.05

Disease progression and treatment timeline are summarized in Fig. 1a. Interestingly, immunohistochemistry staining showed remarkable enhancement of IL-34 expression in Nivolumab-resistant metastatic melanoma compared to melanoma tissues at the primary site (Fig. 1b). To further confirm this, quantification of IL-34 staining on randomly-selected tumor areas in each section was carried out, which showed statistically significant enhancement of IL-34 expression in refractory melanoma tissues (Fig. 2a). Previous reports suggested an important role of IL-34 in the induction of M2-polarized macrophages with immunosuppressive functions from human monocytes [27]. Importantly, cancer cells-derived IL-34 was found to increase frequencies of M2-polarized tumor associated macrophages which showed enhanced immunosuppressive and pro-tumorigenic functions at the tumor microenvironment [23]. In this regard, a recent report has reveals a tumor-associated macrophage-mediated resistance pathway in anti-PD-1 therapy [28]. Consistent with these backgrounds, we found that high expression of IL-34 in refractory metastatic melanoma correlates positively with increased frequencies of CD163^+^ (a marker for M2-polarization) macrophages compared to primary melanoma (Fig. 2b). From these results, we expected an impact for IL-34 expression on prognosis in melanoma patients. Thus, we examined the prognostic impact of *IL34* expression on OS in a cohort of melanoma patients reported by the Human Protein Atlas (http://www.proteinatlas.org) [29]. As expected, Kaplan-Meier analysis of OS showed that high expression of *IL34* significantly correlated with poor prognosis in melanoma (*P* = 0.038).

## Discussion and conclusions

In this study, we report the first case to our knowledge of a patient with a refractory melanoma that showed enhanced expression of IL-34. Our findings described here are consistent with previous reports that suggest the association between IL-34 expression with tumor progression, metastasis, and therapeutic resistance [18–22]. This report further extends the current knowledge regarding the pro-tumorigenic roles of IL-34 in cancer, especially in melanoma that acquired resistance to anti-PD-1 immunotherapy. In this regard, IL-34 may serve as a novel biomarker with prognostic benefits in melanoma patients. IL-34 expression is strongly suggested to correlate with disease stage and poor prognosis in cancers such as brain and lung cancers [23, 26]. Thus, evaluation of IL-34 expression during the treatment course may also help to predict acquired resistance and increased risks of recurrence, and may open new opportunities in prognosis assessment of melanoma patients. From a therapeutic point of view, IL-34 may serve as an important therapeutic target in refractory melanomas. Targeting of IL-34 in chemoresistant lung cancers could help to sensitize chemoresistant tumors to chemotherapy, and enhance anti-tumor immune responses by decreasing frequencies of immunosuppressive macrophages at the tumor microenvironment [23].

Previous studies on the role of IL-34 in cancer have suggested a correlation between IL-34 and acquired resistance to chemotherapy [23–25]. Additionally, high expression of IL-34 associates with disease progression such as in lung cancer, since IL-34 expression in advanced stages (III and IV) was higher than that of early stages (I and II) [25]. In the case of the melanoma patient described here, the high expression of IL-34 was characteristic when chemotherapy and immune checkpoint inhibitor were ineffective in refractory melanoma. Thus, the enhancement of IL-34 expression observed in this case of melanoma patient may accompany acquired resistance to chemotherapy, acquired resistance to immunotherapy, or increased degree of malignancy. Evaluation of IL-34 expression in other clinical samples of melanoma patients should be performed to clarify these issues.

In a remarkable observation, enhanced expression of IL-34 in refractory melanoma was associated with increased frequencies of CD163^+^ macrophages, which have great potential to suppress anti-tumor immunity [23]. Accordingly, IL-34 blockade in IL-34-producing melanomas may help to overcome therapeutic resistance problem, which is under evaluation currently by our team in animal experimental models. In conclusion, we suggest in this report an importance of IL-34 in patients with refractory melanoma, with a great potential as a prognostic biomarker and therapeutic target, which should be evaluated and further extended in future works.

## Abbreviations

CSF1-R: Colony-stimulating factor 1 receptor
CTLA-4: The cytotoxic T-lymphocyte-associated antigen 4
GBM: Glioblastoma
IGF-1: Insulin-like growth factor 1
IL-34: Interleukin-34
IL-4: Interleukin-4
OS: Overall survival
PD: Progressive disease
PD-1: Programmed cell death 1
PR: Partial response
